# Synergistic antiviral activity of *Lactobacillus acidophilus* and *Glycyrrhiza glabra* against Herpes Simplex-1 Virus (HSV-1) and Vesicular Stomatitis Virus (VSV): experimental and *In Silico* insights

**DOI:** 10.1186/s12866-023-02911-z

**Published:** 2023-06-30

**Authors:** Dalia Elebeedy, Aml Ghanem, Shaza H. Aly, Mohamed A. Ali, Ahmed H. I. Faraag, Mohamed K. El-Ashrey, Aya M. salem, Mahmoud A. El Hassab, Ahmed I. Abd El Maksoud

**Affiliations:** 1 Department of Pharmaceutical Biotechnology Faculty of Biotechnology, Misr University for Science and Technology, Giza, Egypt; 2 School of Biotechnology, Badr University in Cairo, Badr City, 11829 Cairo Egypt; 3 Department of Pharmacognosy, Faculty of Pharmacy, Badr University in Cairo, Badr City, Cairo, 11829 Egypt; 4 Botany and Microbiology Department, Faculty of Science, Helwan University, Ain Helwan, Cairo, 11795 Egypt; 5 Department of Pharmaceutical Chemistry, Faculty of Pharmacy, Cairo University, Cairo, Egypt; 6 Department of Medicinal Chemistry, Faculty of Pharmacy, King Salman Inter-National University, Ras Sudr, Egypt; 7 Faculty of Biotechnology, Misr University for Science and Technology, Giza, Egypt; 8 Industrial Biotechnology Department, Genetic Engineering and Biotechnology Research Institute, University of Sadat City, Monufia, Egypt

**Keywords:** Antiviral, Cytotoxicity, HSV-1, *Glycyrrhiza glabra*, *Lactobacillus acidophilus*, Molecular docking, Synergy

## Abstract

**Background:**

The emergence of different viral infections calls for the development of new, effective, and safe antiviral drugs. *Glycyrrhiza glabra* is a well-known herbal remedy possessing antiviral properties.

**Objective:**

The objective of our research was to evaluate the effectiveness of a newly developed combination of the probiotics *Lactobacillus acidophilus* and *G. glabra* root extract against two viral models, namely the DNA virus Herpes simplex virus-1 (HSV-1) and the RNA virus Vesicular Stomatitis Virus (VSV), with regards to their antiviral properties.

**Methodology:**

To examine the antiviral impacts of various treatments, we employed the MTT assay and real-time PCR methodology.

**Results:**

The findings of our study indicate that the co-administration of *L. acidophilus* and *G. glabra* resulted in a significant improvement in the survival rate of Vero cells, while also leading to a reduction in the titers of Herpes Simplex Virus Type 1 (HSV-1) and Vesicular Stomatitis Virus (VSV) in comparison to cells that were not treated. Additionally, an investigation was conducted on glycyrrhizin, the primary constituent of *G. glabra* extract, utilizing molecular docking techniques. The results indicated that glycyrrhizin exhibited a greater binding energy score for HSV-1 polymerase (− 22.45 kcal/mol) and VSV nucleocapsid (− 19.77 kcal/mol) in comparison to the cocrystallized ligand (− 13.31 and − 11.44 kcal/mol, respectively).

**Conclusions:**

The combination of *L. acidophilus* and *G. glabra* extract can be used to develop a new, natural antiviral agent that is safe and effective.

## Introduction

Herpes simplex virus (HSV) is a member of the Herpesviridae family, which consists of a wide variety of enveloped DNA viruses that pose significant global health risks. HSV is a common sexually transmitted infection (STI) that can cause lifelong infections with intermittent recurrence, resulting in painful symptoms and distress for those affected. Moreover, HSV infections can have severe consequences for newborns and immunocompromised individuals, highlighting the need for effective antiviral treatments [1]. According to the World Health Organisation, oral HSV-1 infections affect several billion people, while genital HSV-1 infections affect over 500 million people. This virus places numerous people at risk of various diseases and can cause clinical complications in both adults and neonates [2–4]. The treatment of HSV infection typically involves the use of acyclovir, foscarnet, vidarabine, and other synthetic nucleoside analogues [5, 6], the unintended outcomes of this have led to the emergence of resistant strains of the drug [7, 8]. In addition, the prevention of HSV infection has not been achieved through vaccines. Hence, the focus has shifted towards developing new antiviral drugs and innovative prophylactics in recent decades.

Antiviral properties can be attributed to probiotic bacteria, which generate bacteriocins and lactic acid to inhibit the proliferation of enteric pathogens. Moreover, the interaction of probiotic bacteria with epithelial cell surface receptors can stimulate the production of cytokines, affecting the performance of mucosal lymphocytes [9, 10]. Bioactive secondary metabolites can be sourced from plants and used to develop new natural products with properties such as antiviral, antioxidant, anti-inflammatory, and cytotoxic effects, making them valuable resources in the treatment of various diseases [11–14].

The plant *G. glabra*, also known as licorice from the Fabaceae family, is renowned for its traditional use as an herbal supplement with anti-cancer and anti-viral properties. The roots of this plant are used to treat chronic inflammatory conditions [15, 16].

**Fig. 1:**
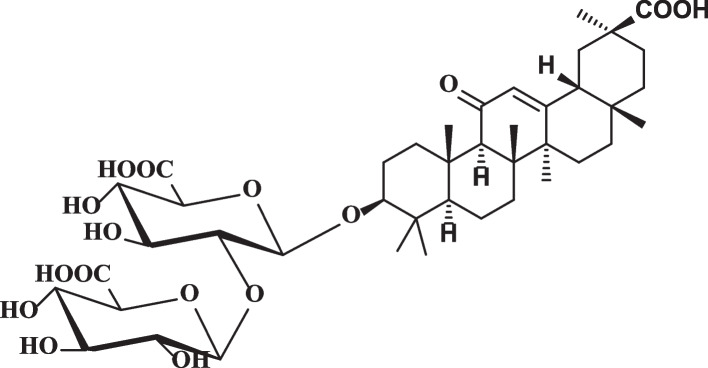
The chemical structure of Glycyrrhizin; the main bioactive compound in *G. glabra*

Secondary metabolites, including terpenoids, saponins, flavonoids, polyamines, and polysaccharides, were found to be abundant in various species of *G. glabra* during chemical analysis [17, 18]. The main active component in *G. glabra* is glycyrrhizin, which belongs to the oleanane category of pentacyclic triterpenoid saponins (as shown in Fig. 1) [17, 19]. The antiviral, anti-inflammatory, antiarrhythmic, antibacterial, antioxidant, and expectorant properties of licorice roots are attributable to glycyrrhizic and aglycone glycyrrhetinic acids present in them [17, 20, 21]. *G. glabra* and its active component, Glycyrrhizin, have been reported to possess antiviral activity against various viruses, including the hepatitis C virus (HCV).

Several studies have demonstrated the potential of glycyrrhizin to inhibit HCV replication in vitro and in vivo. For example, Ikeda et al. (2006) reported that glycyrrhizin inhibited the entry of HCV into hepatocytes by blocking virus binding to cell surface receptors [22]. Similarly, Matsumoto et al. (2013) and Calland et al. (2012) found that glycyrrhizin reduced HCV replication in a dose-dependent manner by inhibiting the expression of HCV RNA-dependent RNA polymerase [23, 24].

In addition to HCV, glycyrrhizin has also been shown to possess antiviral activity against other viruses. For instance, studies have demonstrated the ability of glycyrrhizin to inhibit the replication of influenza viruses [25, 26], herpes simplex viruses [27], and human immunodeficiency viruses [28].

The aim of this study is to assess the potential antiviral effects of combining Lactobacillus acidophilus, a probiotic bacterium, with *G. glabra* against herpes simplex virus type 1 (HSV-1) and vesicular stomatitis virus (VSV), two enveloped viruses that can cause mild to severe illnesses. Previous research has indicated that probiotics possess antiviral properties, while *G. glabra* has been found to have multiple biological activities, including antiviral effects. Thus, this research aims to determine whether the combination of *L. acidophilus* and *G. glabra* can enhance antiviral activity against HSV-1 and VSV.

## Results

### Cytotoxicity

Once the concentrations of probiotics and prebiotics were determined, the Vero cell line underwent a cytotoxicity test, and the microplate reader was used to analyze the results. The the minimum non-toxic concentration (MNTC) for each sample was determined by observing the alterations in cellular morphology.

The cell monolayers were treated with varying amounts of the test substances and left to incubate for 48 hours at 37°C and 5% CO_2_. The determination of non-toxic concentrations was based on a comparison of the treated cell's morphological changes with those of the untreated cells. The MNTC was determined as the maximum concentration that did not cause any damage to the cells.

**Fig. 2:**
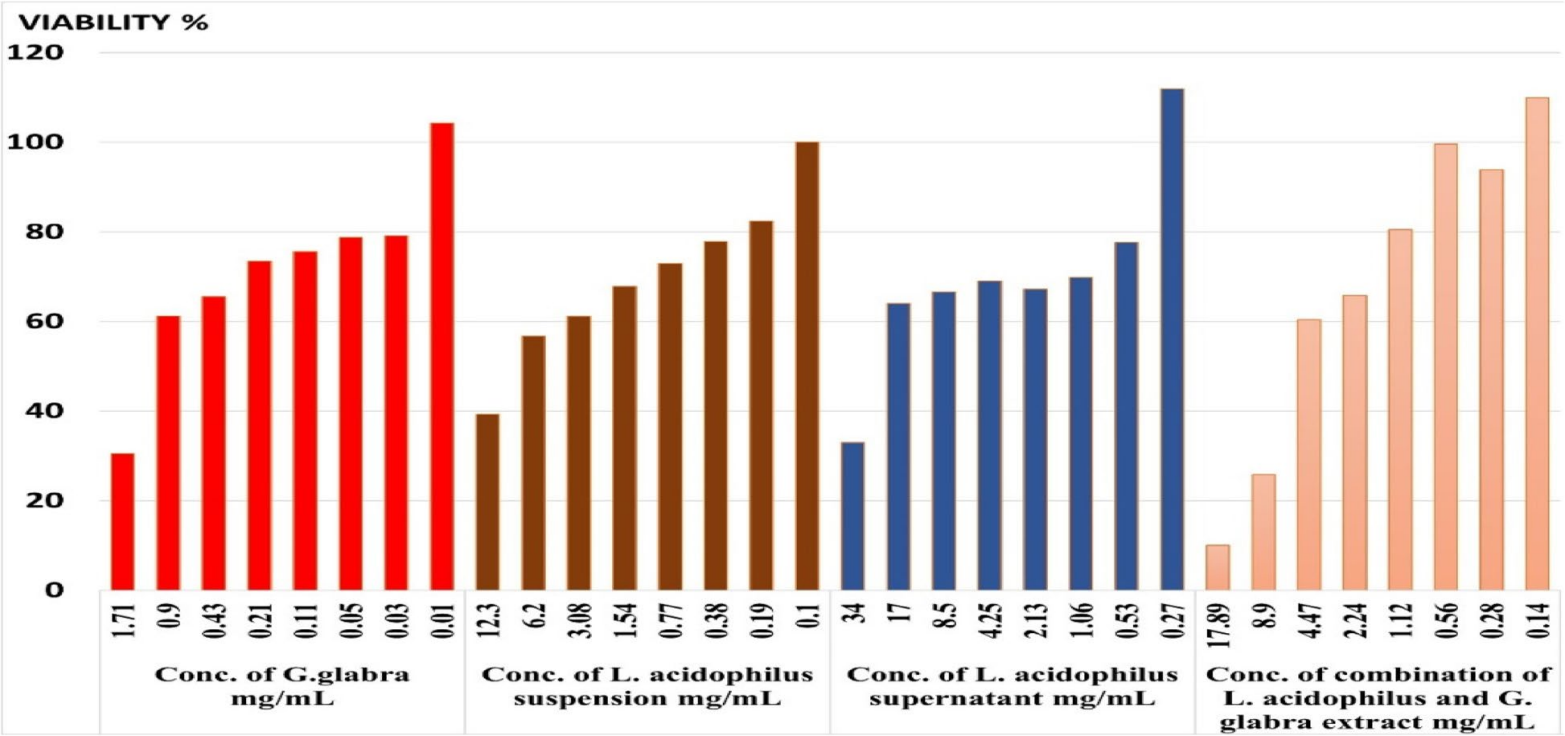
Viability % of Vero cell line after treatment with different concentrations of *G. glabra* extract (**A**), *L. acidophilus* suspension (**B**), *L. acidophilus* supernatant (**C**) and combination of *L. acidophilus* and *G. glabra* extract (**D**)

Once incubated, concentrations of the drug that did not harm live cells were assessed and compared to normal cells to confirm their non-toxicity. Any concentration that led to damaged cell rows was considered hazardous. Additionally, the maximum drug concentrations that had no impact on cells were identified as non-toxic concentrations. By comparing treated and untreated cultures, the minimum non-toxic concentrations (MNTCs) were determined. Table 1 and Figure 2 display the results.

**Table 1:** Viability % of Vero cell line after treatment with different concentrations of *G. glabra* extract, *L. acidophilus* suspension, *L. acidophilus* supernatant and combination of *L. acidophilus* and *G. glabra* extract

Conc. of *G. glabra* mg/ml	Viability %	Conc. of *L. acidophilus* suspension mg/ml	Viability %	Conc. of *L. acidophilus* supernatant mg/ml	Viability %	Conc. of combination of* L. acidophilus* and* G. glabra* extract mg/ml	Viability %
1.71	30.5	12.30	39.3	34.00	33.0	17.89	10.0
0.9	61.2	6.20	56.7	17.00	64.0	8.9	25.8
0.43	65.5	3.08	61.1	8.50	66.6	4.47	60.4
0.21	73.4	1.54	67.8	4.25	69.0	2.24	65.8
0.11	75.6	0.77	72.9	2.13	67.2	1.12	80.5
0.05	78.8	0.38	77.8	1.06	69.8	0.56	99.6
0.03	79.1	0.19	82.4	0.53	77.6	0.28	93.8
0.01	104.3	0.10	100.0	0.27	111.9	0.14	109.9
Working safe conc. mg/mL							
0.9		3.08		17.00		4.47	

(*G. glabra*: 0.9 mg/ml, *L. acidophilus* suspension: 3.08 mg/ml, *L. acidophilus* supernatant: 17.00 mg/ml, and combination of *L. acidophilus* and *G. glabra* extract: 4.47 mg/ml)

### HSV-1 log reduction titre

After establishing the safe concentration of the prebiotic, including *L. acidophilus* suspension and supernatant, and their combination, the cells were exposed to these concentrations. Following this, the cells were inoculated with HSV-1 using varying concentrations obtained through serial dilution of the virus.

**Fig. 3:**
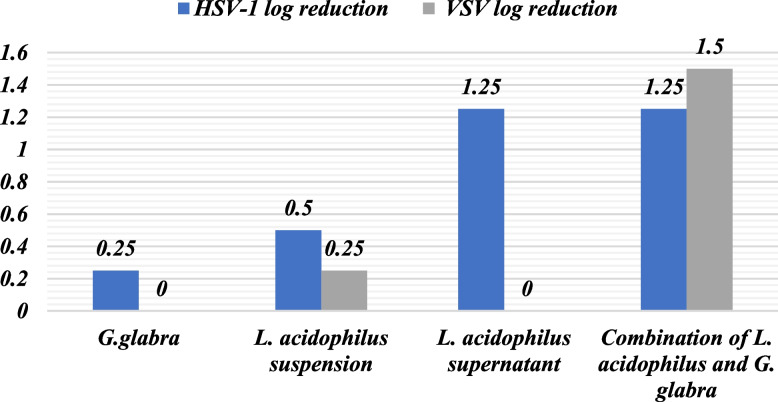
HSV-1 and VSV log reduction in titer after treatment with *G. glabra* extract, *L. acidophilus* suspension, *L. acidophilus* supernatant and combination of *L. acidophilus* and *G. glabra* extract

The Reed and Munch programs were used to determine the log reduction of the virus by comparing its concentration to the initial titer of HSV-1. Table 2 displays the TCID_50_ values for each sample, indicating the log reduction of HSV-1 inhibition, starting from an initial concentration of 1.00E^+07^. The findings are presented in Table 2 and Figure 3.

**Table 2:** HSV-1 and VSV log reduction in titer after treatment with *G. glabra* extract, *L. acidophilus* suspension, *L. acidophilus* supernatant and combination of *L. acidophilus* and *G. glabra* extract

Sample	HSV-1 log reduction	VSV log reduction
*G. glabra*	0.25	0
*L. acidophilus* suspension	0.5	0.25
*L. acidophilus* supernatant	1.25	0
Combination of *L. acidophilus* and *G. glabra*	1.25	1.5

### VSV log reduction titer

The Vero cells were exposed to a non-toxic concentration of the prebiotic, comprising of *L. acidophilus* suspension and supernatant, and their combination. Subsequently, VSV was introduced to the cells at different concentrations, achieved through serial dilution of the virus.

The log reduction of the virus was computed by comparing it to the initial VSV titer using the Reed and Munch programs. The VSV's initial concentration was 1.00E^+05^ TCID_50_, and the resulting virus titer after treatment is presented in Table 2. Each sample was found to inhibit VSV by a certain log reduction, with the results presented in Table 2 and Figure 2.

### Real time PCR

**Fig. 4:**
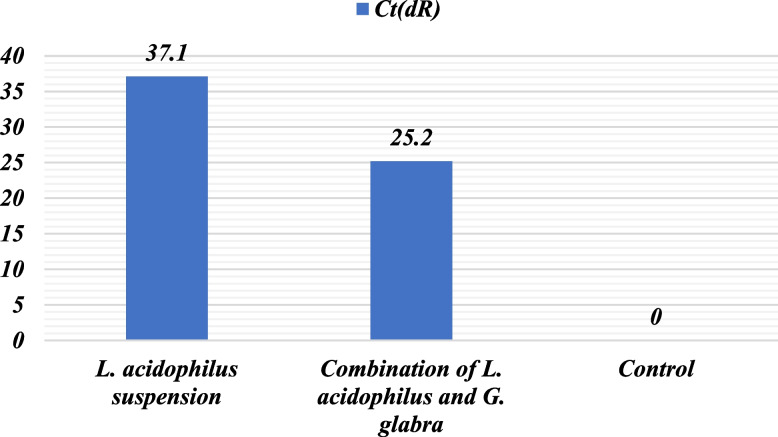
CT ratio showed that combination of *L. acdiophilus* and *G. glabra* achieved better activity than of *L. acdiophilus* suspension alone

Real-time PCR results indicated that the CT ratio of *L. acidophilus* suspension was 37.1 upon exposure to the VSV virus, whereas the combination of *L. acidophilus* and *G. glabra* had a lower CT ratio of 25.2. The control had a CT value of 0. These outcomes imply that the combination of *L. acidophilus* and *G. glabra* could potentially be more efficacious in hindering the VSV virus than *L. acidophilus* suspension alone, as presented in Table 3 and Figure 4.

**Table 3:** CT ratio of samples on VSV virus

Sample	CT (dR)
*L. acidophilus* suspension	37.1
Combination of *L. acidophilus* and *G. glabra*	25.2
Control	0

### *In silico* studies

Molecular docking investigations were conducted to investigate and predict the possible biological targets of glycyrrhizin, including HSV-1 and VSV. The binding energy scores of glycyrrhizin were compared to those of the co-crystallized ligands, and the results are presented in Table 4.

**Table 4:** Binding energy scores for glycyrrhizin

Virus	Target	PDB	Co-crystallized ligand score (kcal/mol.)	Glycyrrhizin score (kcal/mol.)
**HSV-1**	Terminase	6M5U	-14.77	-9.29
	Glycolase	2C53	-12.58	-12.71
	Thymidine Kinase	2KI5	-11.54	.-13.98
	Polymerase	7LUF	-13.31	-22.45
**VSV**	Nucleocapsid	6BJY	-11.44	-19.77
	L-Protein	4UCZ	-24.07	-17.96

**Fig. 5:**
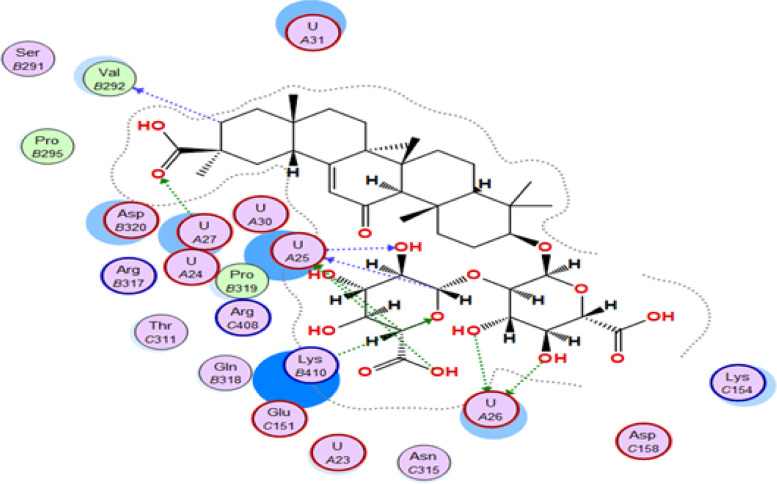
2D interaction of glycyrrhizin with HSV-1 Polymerase binding site

**Fig. 6:**
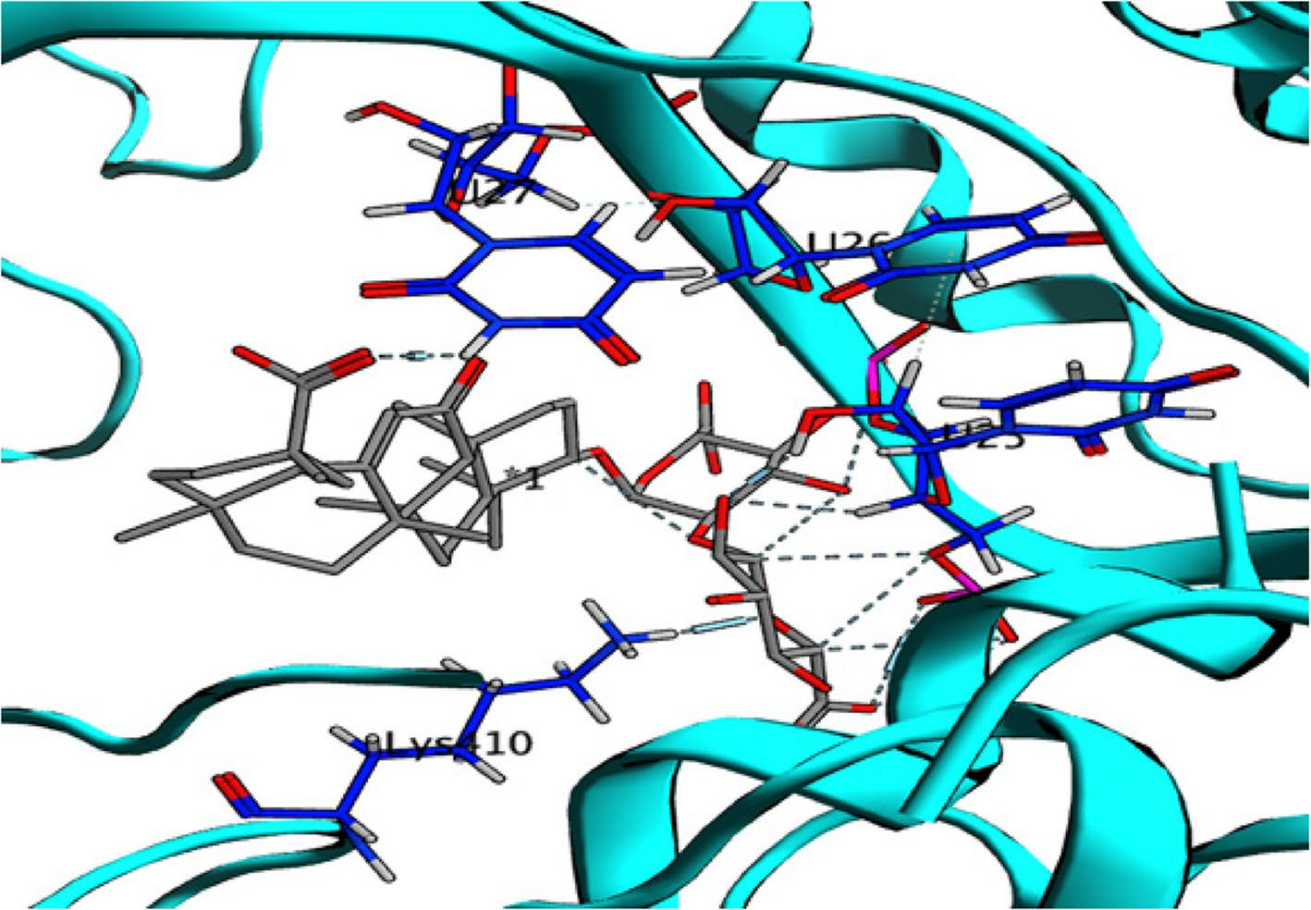
3D interaction of glycyrrhizin with HSV-1 Polymerase binding site

In Table 4, it is evident that glycyrrhizin exhibits a remarkable energy binding score (-22.45 kcal/mol) against the HSV-1 polymerase enzyme, which is notably higher than the energy score of the co-crystallized ligand (-13.31 kcal/mol). The interaction of glycyrrhizin with crucial amino acids in the enzyme is facilitated through conventional hydrogen bonds, which involves its oxygen atom with Asp717 and Lys928, its carboxylic OH group with Val812, Gln617, Ser816, and Gly819, as well as the guanine base of DG-10, as illustrated in Figures 5 and 6 [29]. These findings indicate a higher likelihood of glycyrrhizin serving as an inhibitor of HSV-1 polymerase. The results imply that glycyrrhizin is more likely to act as a HSV-1 polymerase inhibitor because of its high energy binding score and the precise amino acid interactions it establishes with the enzyme.

**Fig. 7:**
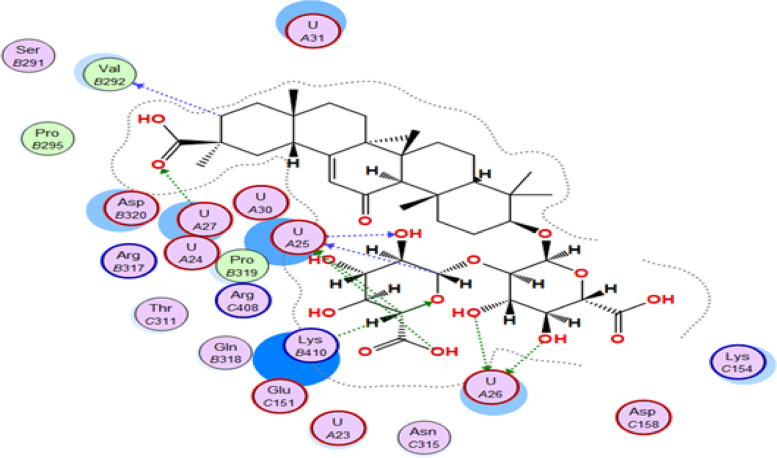
2D interaction of glycyrrhizin with VSV nucleocapsid binding site

**Fig. 8:**
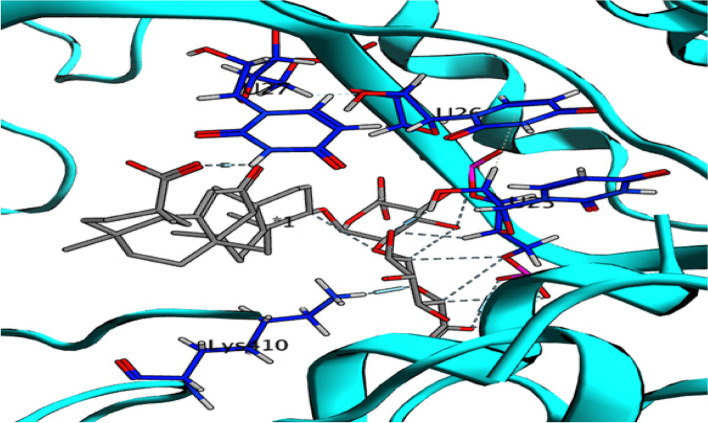
3D interaction of glycyrrhizin with VSV nucleocapsid binding site

The binding affinity between glycyrrhizin and VSV nucleocapsid was found to be significant, with a score of -19.77 kcal/mol, surpassing that of the co-crystallized ligand, which had a score of -11.44 kcal/mol. Glycyrrhizin binds to uridine (U25) via its OH group and to uridine (U26) via two other hydroxyl groups, as shown in Figures 7 and 8. Furthermore, the carboxylic acid's carbonyl oxygen of glycyrrhizin interacts with uridine (U27), and the Lys410 amino acid interacts with the pyran oxygen [30]. Based on the significant interactions and high binding score, it can be inferred that glycyrrhizin has the ability to hinder the activity of VSV nucleocapsid.

## Discussion

Phenolic compounds and triterpene saponins are a diverse collection of natural compounds found in plants that possess a range of biological activities, such as antiviral effects. Phenolic compounds have been found to exhibit antiviral properties against various viruses such as herpes simplex virus, influenza virus, and human immunodeficiency virus, among others, according to multiple studies. Phenolic compounds have the ability to hinder viral attachment and entry into host cells. Caffeic acid, which is a common phenolic acid, inhibits the entry of herpes simplex virus type 1 (HSV-1) into Vero cells by possibly interacting with the viral envelope glycoproteins [29]. The inhibition of hepatitis C virus (HCV) entry into Huh-7.5 cells can be attributed to quercetin, a flavonoid present in various fruits and vegetables. This is believed to occur through the obstruction of the interaction between the virus and its receptor on the surface of the cell [30]. Studies have demonstrated that phenolic compounds can hinder viral replication and assembly. Resveratrol, a type of polyphenolic compound present in red wine and grapes, has been found to impede the replication of three strains of influenza virus both in vitro and in vivo, by potentially disrupting the activity of the viral polymerase [31]. Furthermore, the assembly and release of HCV virions from infected cells were hindered by gallic acid, which is another prevalent phenolic acid [32–34]. In addition, it has been reported that phenolic compounds can alter the immune response of the host to viral infections. One example is curcumin, a phenolic compound present in turmeric, which has been demonstrated to boost the antiviral effects of interferon-alpha (IFN-α) against dengue virus in vitro by increasing the expression of IFN-stimulated genes [35]. In the same way, it was discovered that epigallocatechin gallate (EGCG), a main catechin present in green tea, boosted the effectiveness of IFN-α against hepatitis B virus (HBV) by increasing the expression of IFN-stimulated genes in human hepatoma cells [35]. Phenolic and triterpene compounds are a promising class of natural compounds that have antiviral activity against a wide range of viruses. Their mechanism of action involves inhibiting viral attachment and entry, replication and assembly, and modulation of the host immune response.

Glycyrrhizin, extracted from the roots of the licorice plant, is often misconceived as a phenolic compound, but in reality, it is a triterpene saponin. Research has been conducted on its antiviral and anti-inflammatory properties, specifically its ability to hinder the replication of several viruses including hepatitis C virus, HIV-1, and SARS-associated coronavirus (SARS-CoV) [36–43].

The aim of the study was to analyze the separate impact of *L. acidophilus* as a probiotic and *G. glabra* extract as a prebiotic, as well as their combined effect as a symbiotic, on the Vero cell line that was infected with HSV-1 and VSV viruses. Although *L. acidophilus* and *G. glabra* were given to the infected Vero cells, the exact mechanism through which *L. acidophilus* fights viral infections *L. acidophilus* fights viral infections by blocking the pathogen's interaction with DC-specific intercellular adhesion molecule 3-grabbing non-integrin (DC-SIGN), a trans-membrane protein, through the surface layer protein (S-layer) present on the bacterial cell-envelope of *L. acidophilus*. By doing so, it inhibits viral and bacterial infections [44]. The S-layer protein of *L. acidophilus* is also shown to have antiviral activity by impacting virus infections and virus-induced apoptosis [45]. Additionally, healthy bacteria like *L. acidophilus* can boost the immune system and help reduce the risk of viral infections [46, 47], the byproducts of metabolic activity by *L. acidophilus*, such as bacteriocins, lactic acid, and H_2_O_2_, could potentially aid in preventing virus proliferation and improving the innate immune response [48, 49], this is supported by previous research indicating that the metabolites generated by *L. acidophilus* can promote the production of cytokines, which are crucial in the fight against viral infections such as COVID-19 [50–54]. Additionally, bacteriocins produced by *L. acidophilus* have been shown to hinder the replication of certain viruses, such as herpes simplex virus and human papillomavirus [55].

The introduction of *L. acidophilus* and *G. glabra* resulted in a noteworthy decrease in viral multiplication, as demonstrated by the MTT assay conducted on a microplate reader and real-time PCR. These tests indicated a reduction in the expression rate on the MX gene. Earlier studies have confirmed that *G. glabra* exhibits anti-HSV1 activity, with an IC_50_ value of 225 ± 24.1 µM [56]. The immunoregulatory and anti-inflammatory properties of *G. glabra* can impede the entry of HSV-1 virus into cell [29, 57].

The study demonstrated that the combination of *L. acidophilus* and *G. glabra* is a powerful antiviral agent, showing a greater reduction in viral titer than either treatment alone. Additionally, the presence of *L. acidophilus* and *G. glabra* significantly increased the viability of the Vero cell line both before and after infection with HSV-1. These results suggest that the combination of these probiotic and prebiotic agents can improve Vero cell viability against viral infections and decrease viral titers. The study also indicates that the combined treatment is more effective than either one used alone.

Healthy women's vaginal discharge was the source of the samples, as reported by Goudarzi and Fazeli (2015) [58].

The present strains of *Lactobacilli* were differentiated using biochemical and molecular analysis, and the antiviral efficacy of the culture supernatant (CS) of *L. acidophilus* species LA-5 was tested against the standard strain of HSV-1. Recent research has indicated that the *L. acidophilus* culture supernatant has a noteworthy capability to decrease the formation of HSV-1 and HIV plaques on cells [59].

Notably, the inhibitory effect is observed in both the standard strain and the wild *L. acidophilus*, without the need for an acidic environment or the production of H_2_O_2_ or H^+^ ions, suggesting that active metabolites may be responsible. With an estimated 50 million people in the US infected with genital herpes through direct contact and up to 60% of sexually active adults carrying the virus, the use of *L. acidophilus* for controlling HSV-1 is a significant possibility. It is important to note that the virus can spread through asymptomatic carriers, leading to high transmission rates [60].

Our study demonstrated a significant decrease in HSV-1 replication using a supernatant culture of *L. acidophilus*. This finding suggests that lactobacilli possess a potent antiviral capacity, potentially through the production of bacteriocins or other fragments that can neutralize the virus. This mode of antiviral activity differs from previously reported mechanisms that involve the secretion of H_2_O_2_ and H^+^ ions [55]. The culture supernatant (CS) utilized in our study had a neutral pH and did not exhibit any toxic effects on the target cells. This may be attributed to the release of active molecules in the CS of the *L. acidophilus* strain, which exerted a significant inhibitory effect on the replication of the virus [55, 61, 62].

In our study, the antiviral defense mechanism displayed by *L. acidophilus* did not involve the generation of H2O2 or H+ ions. Rather, it is plausible that the byproducts produced by the lactobacilli impeded the attachment of HSV virions to host cells or counteracted the viral particles. This mechanism is a recent proposal and contradicts the previous notion that lactobacilli deactivate viral particles by reducing pH levels or discharging disinfectant agents [63].

Several research studies have explored the antiviral mechanism of different treatments for viruses like HSV-1, VSV, and MERS-CoV, and their correlation with the Mx-A gene's expression profiles. In one such study, it was observed that the MxA protein obstructed the transcription of VSV, which aligns with the inhibitory properties of these treatments against VSV [64].

In addition, it has been observed that the MxA protein produced in *Escherichia coli* can hinder the RNA synthesis of VSV and influenza A virus in vitro [65]. Other investigations have also highlighted the capability of MxA protein to impede thogoto virus [64], bunya, phlebo, and hanta viruses [66], as well as La-Crosse virus [67], and puumala and tula hantaviruses [65].

The inhibitory effect of MxA protein on DUGV replication was observed to be due to its ability to decrease the expression levels of DUGV antigen, as per the findings of [68]. However, the antiviral activity of MxA protein is dependent on the type of virus and host cell. For instance, MxA protein demonstrated inhibitory effects on measles virus in human mononuclear and glioblastoma cell lines but not in Vero or Hep-2 cells [69]. In U87 or Vero cells, MxA protein did not inhibit respiratory syncytial virus (RSV) [55]. Nevertheless, transgenic mouse cells that expressed bovine Mx protein exhibited complete abolition of murine pneumovirus infectivity [70].

## Conclusions

The study findings indicate that probiotics and prebiotics have antiviral effects against HSV-1 and VSV, as shown by MTT assay and real-time PCR. The combination of *L. acidophilus* and *G. glabra* was observed to enhance cell survival against viral infection, possibly by hindering intracellular viral reproduction or interfering with early infection stages. The study also revealed that glycyrrhizin, the primary component in *G. glabra* extract, exhibits high binding energy scores against HSV-1 polymerase enzyme and favorable interactions with VSV nucleocapsid. Combining *L. acidophilus* and *G. glabra* extract could lead to the development of a natural antiviral agent that is both safe and effective. The synergistic effect of *L. acidophilus* and glycyrrhizin makes them a suitable combination for viral therapy. Further research is needed to investigate the potential of the major bioactive ingredient and its antiviral efficacy in developing a viable natural antiviral medication against HSV-1 and VSV.

## Methods

### Preparation of G. glabra plant extract

Licorice roots harvested from Afghanistan were procured from an Egyptian market and ground to a fine powder in a Waring blender. Water extraction of the root samples was conducted for 20 hours to prevent microbial growth that could result from prolonged soaking in cold water. The extraction process was performed only once, and the resultant extract was filtered using linen filter cloth and concentrated via a rotary evaporator. The concentrated residue was stored in dark, sealed glass vials at a temperature of -20°C for future analysis.

### Bacterial strain

The lyophilized strain of the probiotic *L. acidophilus* was procured from the American Type Culture Collection (43121) and grown in Man, Rogosa, and Sharpe (MRS) media (Acumedia, United States) at 37°C with 180 rpm shaking overnight. The *L. acidophilus* was isolated from a fresh MRS culture by centrifugation at 3000 rpm, and the pellet was washed twice with phosphate-buffered saline (PBS) to remove any residual MRS. The supernatant was filtered through a 0.2 m membrane. The bacterial suspension from an overnight culture was sonicated at 60% intensity using bacterial suspension aliquots (Fisher Scientific Co., NJ, USA) with the help of the Sonic 300 Dismembrator's intermediate sonication horn. The horn was inserted into a 15-ml polypropylene tube with 5–6 ml of cell solution, measuring approximately 9.5 mm in diameter, which was placed in an ice water bath to cool while sonicating the samples.

### In vitro antiviral study

#### Test viruses

The DNA virus model HSV-1 and the RNA virus model VSV Indiana strain-156 were generously provided by the International Center for Training and Advanced Research in Egypt.

#### Cell line and growth conditions

The Vero cell line (ATCC no. CCL-81), derived from African green monkeys, was provided by VACSERA Egypt for this study. The cells were cultured in Dulbecco's Modified Eagle Medium (Sigma) supplemented with 5% fetal bovine serum (FBS, Sigma) and incubated in a humidified atmosphere with 5% CO_2_ at 37°C (Jouan-France) for 48 hours in 96-well plates to attain a confluent monolayer. The culture medium used for the Vero cells contained 10% heat inactivated FBS, 100 U/ml penicillin, 100 μg/ml streptomycin, and 2 mM/ml glutamine, all procured from Sigma Aldrich in the United States.

#### Cytotoxicity assay

The evaluation of cytotoxicity was carried out by observing changes in the cellular morphology, as previously explained [71]. The samples were distributed into four Falcon tubes: the first tube was given 300 μl of the prebiotic (*G. glabra* extract), the second was given 300 μl of the probiotic *L. acidophilus* (cell sonicate), the third was given 300 μl of the probiotic *L. acidophilus* (cell supernatant), and the fourth tube was given a mixture of the prebiotic and probiotic (100 μl of *G. glabra* extract + 100 μl of *L. acidophilus* cell supernatant + 100 μl of cell precipitate). Vero cells were seeded onto a 96-well ELISA plate, followed by incubation at 37°C in a 5% CO_2_ atmosphere for 24 hours. Another sterile 96-well plate was labeled and divided into three columns for each sample. Samples were pipetted into the first row of the plate (200 μl each) and then subjected to twofold dilution. Subsequently, 100 μL of Vero cells were added to each well, and the plate was incubated at 37°C for 24 hours. After incubation, the medium was discarded, and the monolayer was washed with PBS (pH 7.4).

#### MTT assay

To determine the cytotoxic effects of prebiotics, probiotics, and combination treatments, various concentrations were tested using the 3-(4,5-dimethylthiazol-2-yl)-2,5-diphenyltetrazolium bromide (MTT) assay. The initial concentration of treatment was 100 mg/ml, followed by two-fold serial dilutions in serum-free DMEM (100 L of each dilution/well). Treated Vero cells were incubated at 37°C for 24 hours, washed thrice with 250 μl of PBS, and 50 μl of MTT solution (0.5 mg/ml) was added to each well. After incubating the plates at 37°C for four hours, the formed, purple-colored formazan crystals were dissolved in 50 μl of dimethyl sulfoxide (Sigma Aldrich, USA) after washing the plates with PBS once more. The plates were incubated on a shaker at room temperature for 5 min, and the optical density (OD) was measured at 570 nm using an ELISA plate reader. The percentage of cellular viability was calculated using the given formula:

$$\text{Viability} (\%) = \left( \frac{\text{Mean OD of test wells}}{\text{Mean OD of control wells}} \right) \times 100 $$

#### Preparation of viral assay

To assess the viral infectivity, Vero cells were exposed to 10-fold serial dilutions of HSV-1 and VSV, each added to eight wells. The plates were incubated at 37°C for seven days and examined daily for cytopathic effects using a microscope from Hund, Germany. The median tissue culture infectious dose (TCID_50_) was determined using the Reed and Muench method [60].

To evaluate the direct antiviral effects, Vero cells were infected with serially diluted HSV-1 or VSV for one hour at 37°C. After removing unabsorbed viruses, the cells were washed thrice with PBS. The infected cells were then treated with safe concentrations of the prebiotic, probiotic, or their combination and incubated for 24 hours at 37°C. The cells were examined microscopically for cytopathic effects [55].

Cells were treated with safe levels of the formulas for 24 hours at 37°C to evaluate their indirect antiviral activity. After removing the treatment medium, Vero cells were infected with a series of viruses diluted 10-fold [62].

Using established methods, the reduction in virus infectivity titer was measured as a percentage and compared between treated and untreated plates using both techniques.

#### Virus titration calculation of TCID_50_

Reed and Muench's method were used to calculate the inhibition of virus titer. The log reduction of the initial viral titer was then determined by subtracting the titer after treatment from the initial titer [72, 73].

#### RNA extraction from cell lines

Real-time PCR was used to evaluate the levels of MX gene expression. Total RNA was extracted from Vero cells that were either untreated or treated with prebiotic, probiotic, or their combination, using the GeneJET RNA Purification kit (Fermantus, UK) according to the manufacturer's protocol. The RNA's quality and quantity were assessed by measuring its absorbance at 260 and 280 nm. The Quantitect Reverse Transcription kit (Qiagen, Germany) was used to generate cDNA from 1 μg of the extracted RNA. The expression levels of the MxA gene were measured using the following primers:

In order to amplify the Mx-A gene, the primers F (5'-AAA TGG CTC AAG AGG TGGA-3') and R (5'-TAT CGC TGA CAG TTG GGTG-3') were utilized, and melting curves were created to confirm the successful amplification of the desired product. Additionally, a standard curve was generated to evaluate the amplification efficiency and relative alterations in expression levels [74].

### *In silico* studies

The use of molecular docking in computer-assisted drug design has been widespread in recent times, as it aids in predicting binding affinities and analyzing the interactions between receptors and ligands [75]. For this study, the Molecular Operating Environment (MOE, version 2019.0102) software was employed [76] to execute all docking procedures. The 2D structure of glycyrrhizin was drawn using ChemDraw and transformed into a 3D structure using MOE. All ligands, including those that were co-crystallized, were subjected to energy minimization with the MMFF94x force field until a root mean square deviation gradient of 0.1 kcal mol^-1^Å^-1^ was achieved [77].

The X-ray crystallographic structures of the most important targets of HSV-1 were retrieved from Protein Data Bank with the following IDs: 6M5U (terminase), 2C53 (glycolase), 2KI5 (thymidine kinase), and 7LUF (polymerase). Nucleocapsid (6BJY) and L-protein (4UCZ) were chosen as possible targets for VSV.

To identify the binding site, the cocrystallized ligand present in each protein file was utilized. Docking was performed by employing Triangle Matcher as a placement technique and London dG as a scoring algorithm. The output of the docking was visualized using MOE software to create 2D and 3D images.

## Data Availability

The data generated during this study are available from the corresponding author upon reasonable request.

## Abbreviations

VSV: Vesicular stomatitis virus
HSV-1: Herpes simplex virus-1
MNTC: Minimum non-toxic concentration
CID50: Tissue culture infectious dose 50
CT: Cycle threshold
